# Draft Genome Sequence of *Achromobacter* sp. Strain AR476-2, Isolated from a Cellulolytic Consortium

**DOI:** 10.1128/genomeA.00587-16

**Published:** 2016-06-23

**Authors:** Daniel Kurth, Cintia M. Romero, Pablo M. Fernandez, Marcela A. Ferrero, M. Alejandra Martinez

**Affiliations:** a Planta Piloto de Procesos Industriales Microbiológicos (PROIMI), CONICET, Tucumán, Argentina; b Facultad de Bioquímica, Química y Farmacia, Universidad Nacional de Tucumán, Tucumán, Argentina; c Facultad de Ciencias Exactas y Tecnología, Universidad Nacional de Tucumán, Tucumán, Argentina

## Abstract

*Achromobacter* sp. AR476-2 is a noncellulolytic strain previously isolated from a cellulolytic consortium selected from samples of insect gut. Its genome sequence could contribute to the unraveling of the complex interaction of microorganisms and enzymes involved in the biodegradation of lignocellulosic biomass in nature.

## GENOME ANNOUNCEMENT

*Achromobacter* sp. AR476-2 is a Gram-negative isolated from a cellulolytic bacterial consortia selected from the guts of *Diatraea saccharalis* larvae (1). Isolates belonging to this genus were described as opportunistic pathogens (2) and the potential for environmental bioremediation applications has been recently targeted (3). The genomic analysis showed that AR476-2 strain is closely related to *A. piechaudii* strain HLE (4) and to *A. xylosoxidans* A8 (5).

The genome sequencing of the strain AR476-2 was performed using two lanes in an Illumina HiSeq 1500, producing 20,525,460 paired-end reads (2 × 100 bp) with a median sequencing coverage of 438×. Quality filtering and trimming was performed with Perl script (6). A complete quality control analysis with Fast QC was also performed on the resulting reads (http://www.bioinformatics.babraham.ac.uk/projects/fastqc/). Paired-end reads were assembled into 61 scaffolds with the A5 assembler (7). Automatic annotation using the RAST (8) web server was carried out to for initial characterization and the submitted genome was annotated by the NCBI Prokaryotic Genome Annotation Pipeline (http://www.ncbi.nlm.nih.gov/genome/annotation_prok/).

*Achromobacter* sp. AR476-2 has a chromosome size of 6,515,255 bp, a G+C content of 65.1% and contains 6,053 putative coding sequences (CDSs) (964-bp average lengths). The project accession also contains sequences for 68 RNA loci.

The ability to use complex carbohydrates has not been described for members of *Achromobacter* spp. However, isolates belonging to this genus have been found to be associated with cellulolytic bacterium; *Achromobacter* sp. CX2 was described as an extracellular β-glucosidase producing bacterium that shows enzymatic synergism with cellulolytic bacteria (9). In agreement with this, the annotation revealed that the genome of AR476-2 does not contain genes encoding hydrolases responsible for the cellulose or xylan degradation, although an α-amylase belonging to GH13 family was detected, associated with modules of glycogen-binding function CMB48. The endurance of *Achromobacter* spp. within lignocellulosic consortia could be due to their ability to utilize simple sugars produced by potent hydrolytic strains (9, 10). The *Achromobacter* sp. AR476-2 genome revealed the presence of catalytic modules of β-glucosidases and β-xylosidases (11). However, the role of noncellulolytic strains as a part of the lignocellulosic consortia in nature needs to be further studied.

### Nucleotide sequence accession numbers.

The *Achromobacter* sp. AR476-2 whole-genome shotgun (WGS) project has the project accession no. LWDT00000000. This version of the project (01) is LWDT01000000.
